# Supplemental Xylooligosaccharide Modulates Intestinal Mucosal Barrier and Cecal Microbiota in Laying Hens Fed Oxidized Fish Oil

**DOI:** 10.3389/fmicb.2021.635333

**Published:** 2021-02-22

**Authors:** Jian-min Zhou, Hai-jun Zhang, Shu-geng Wu, Kai Qiu, Yu Fu, Guang-hai Qi, Jing Wang

**Affiliations:** Laboratory of Quality and Safety Risk Assessment for Animal Products on Feed Hazards (Beijing) of the Ministry of Agriculture and Rural Affairs, Feed Research Institute, Chinese Academy of Agricultural Sciences, Beijing, China

**Keywords:** oxidized fish oil, laying hen, intestinal mucosal barrier, gut microbiota, XOS

## Abstract

Our previous study indicated that dietary xylooligosaccharide (XOS) supplementation improved feed efficiency, ileal morphology, and nutrient digestibility in laying hens. The objective of this study was to evaluate the mitigative effects of XOS on intestinal mucosal barrier impairment and microbiota dysbiosis induced by oxidized fish oil (OFO) in laying hens. A total of 384 Hy-Line Brown layers at 50 weeks of age were randomly divided into four dietary treatments, including the diets supplemented with 20 g/kg of fresh fish oil (FFO group) or 20 g/kg of oxidized fish oil (OFO group), and the OFO diets with XOS addition at 200 mg/kg (OFO/XOS_200_ group) or 400 mg/kg (OFO/XOS_400_ group). Each treatment had eight replicates with 12 birds each. The OFO treatment decreased (*P* < 0.05) the production performance of birds from 7 to 12 weeks of the experiment, reduced (*P* < 0.05) ileal mucosal secretory immunoglobulin A (sIgA) content, and increased (*P* < 0.05) serum endotoxin concentration, as well as downregulated (*P* < 0.05) mRNA expression of claudin-1 (*CLDN1*) and claudin-5 (*CLDN5*) in the ileal mucosa at the end of the experiment. Dietary XOS addition (400 mg/kg) recovered (*P* < 0.05) these changes and further improved (*P* < 0.05) ileal villus height (VH) and the villus height-to-crypt depth ratio (VCR). In addition, OFO treatment altered cecal microbial composition of layers, and these alterations were probably involved in OFO-induced ileal mucosal impairment as causes or consequences. Supplemental XOS remodeled cecal microbiota of layers fed the OFO diet, characterized by an elevation in microbial richness and changes in microbial composition, including increases in *Firmicutes*, *Ruminococcaceae*, *Verrucomicrobia* (*Akkermansia*), *Paraprevotella*, *Prevotella_9*, and *Oscillospira*, along with a decrease in *Erysipelatoclostridium.* The increased abundance of *Verrucomicrobia* (*Akkermansia*) had positive correlations with the improved ileal VH and ileal mucosal expression of *CLDN1*. The abundance of *Erysipelatoclostridium* decreased by XOS addition was negatively associated with ileal VH, VCR, ileal mucosal sIgA content, and the relative expression of zonula occludens-2, *CLDN1*, and *CLDN5*. Collectively, supplemental XOS alleviated OFO-induced intestinal mucosal barrier dysfunction and performance impairment in laying hens, which could be at least partially attributed to the modulation of gut microbiota.

## Introduction

The intestinal mucosa is a dynamic interface and mainly consists of epithelial cells linked by tight junctions, separating hostile external elements from internal milieu (Sánchez de Medina et al., 2014). Functionally, the intestinal mucosal barrier not only guarantees nutrient absorption but also ensures adequate containment of detrimental luminal contents through the turnover of mucus, production of secretory immunoglobulin A (sIgA), and maintenance of tight junctions (Derrien et al., 2017; Xie et al., 2019a). However, some harmful ingredients in laying hen diets can damage the intestinal mucosal barrier and further elicit translocation of pathogens, progression of inflammation, and dysfunction of nutrient absorption, resulting in poor performance and economic efficiency (Xing et al., 2019; Miao et al., 2020). Fish oil rich in *n*-3 polyunsaturated fatty acids (PUFA) has been widely added to layer diets to produce *n*-3 PUFA-enriched eggs (Dong et al., 2018; Feng et al., 2019). Nevertheless, high *n*-3 PUFA content in fish oil also means that it is easily oxidized, generating primary and secondary lipid peroxidation products. These toxicants such as lipid hydroperoxides are toxic substances that can damage the intestinal epithelial cells by reducing membrane integrity and disturbing membrane permeability of birds (Dibner et al., 1996; Dasilva et al., 2017). Tan et al. (2019) demonstrated that supplementation of broiler diets with 4% oxidized fish oil (OFO) decreased the expression of tight junction proteins and upregulated the expression of pro-inflammatory cytokine *IL-22* in the intestinal mucosa. Previous publications have shown that the use of antioxidants such as ascorbic acid and polyphenols are effective in preventing lipid oxidation during gastrointestinal digestion (Lapidot et al., 2005; Maestre et al., 2013). However, there is limited information concerning the repair of intestinal mucosal barrier induced by oxidized lipids.

Xylooligosaccharide (XOS) is a sugar oligomer of two to seven xylose units linked by β-1,4-glycosidic bonds (Jordan and Kurt, 2010). Although chickens lack enzymes required to degrade the glycoside links between xylose units, XOS is available for fermentation in the cecum by xylanolytic bacteria (Pourabedin and Zhao, 2015). Selective fermentation of XOS in turn shifted the gut microbiota toward a relative increase in probiotics and a decrease in pathogenic bacteria, thus contributing to the improvements in intestinal health and immune function in poultry (De Maesschalck et al., 2015; Ding et al., 2018; Lin et al., 2018). It was reported that XOS addition enhanced the intestinal barrier function by upregulating the expression of tight junction protein occludin in rats (Christensen et al., 2014). Similarly, dietary supplementation of prebiotics and synbiotics (both contained 150 mg/kg XOS) improved small intestinal morphology and sIgA content in broilers (Min et al., 2016). Besides, XOS was also demonstrated to mitigate high-fat diet-induced intestinal inflammation and barrier dysfunction by modulating gut microbial composition (Fei et al., 2020). Accordingly, addition of XOS may exert active roles in protecting intestinal function in chickens. The current study was aimed to investigate the positive effects of XOS addition on gut microbiota and intestinal mucosal barrier in laying hens fed OFO.

Gut microbiota composition is closely linked to the intestinal barrier function, including the modulations of epithelial shield, mucus layer, and mucosal immunity (Alam and Neish, 2018). Although our previous study has identified that XOS addition can improve feed efficiency and ileal morphology, and modulate cecal microbial composition (Zhou et al., 2020), it is still unknown whether XOS addition can protect intestinal mucosal barrier from damage through regulating gut microbiota dysbiosis. In keeping with this, the objective of this study was to assess the alteration of gut microbiota to explain the possible mechanism through which XOS alleviates OFO-induced impairment of intestinal mucosal barrier.

## Materials and Methods

### Ethics Statement

All experimental protocols were approved by the Animal Care and Use Committee of the Feed Research Institute of the Chinese Academy of Agricultural Sciences (ACE-CAAS-20180915), and the methods were carried out in accordance with the relevant guidelines and regulations.

### Fish Oil Preparation

Fresh fish oil (FFO) without any antioxidant was purchased from the Foshan Fish Oil Production Company (Guangdong, China) and stored in a refrigerator at −20°C prior to their addition to feed. The oxidized fish oil was prepared by heating the fresh fish oil at 90°C with vigorous aeration for 72 h. Peroxide values of fresh and oxidized fish oil analyzed according to the ISO method ISO 3960 (International Organization for Standarization [ISO], 2001) were 3.20 and 22.25 meq/kg, respectively.

### Birds and Experimental Design

A total of 384 Hy-Line Brown layers aged 50 weeks were randomly allocated into four groups, with eight replicates (12 birds in four adjacent cages as a replicate). Initial body weight and egg production were similar across all the replicates. The treatment groups were as follows: FFO group, birds were fed diet with 20 g/kg fresh fish oil; OFO group, birds were fed diet with 20 g/kg of oxidized fish oil; OFO/XOS_200_ group, birds were fed diet with 20 g/kg of oxidized fish oil, and 200 mg/kg of XOS; and OFO/XOS_400_ group, birds were fed diet with 20 g/kg of oxidized fish oil and 400 mg/kg of XOS. Laying hens for the trial were allocated to three-tier battery cages of three laying hens each (cage size: 40 cm × 40 cm × 35 cm) and exposed to 16 h of light/day with an intensity of 20 lx. Temperature of layer house was affected by seasonal change, which was pretty higher in the first 6 weeks (between 27 and 32°C) than that in the last 6 weeks (between 24 and 28°C). Diets and water were offered *ad libitum* in mash form and by nipple drinkers, respectively. All hens remained in good health during the feeding period. There were no culled birds, and medical intervention was not applied to any bird. The experimental diet (Table 1) was formulated according to the National Research and Council (1994). The XOS (XOS95P) added in the diets was purchased from a commercial supplier (Jinan Longlive Biology Co., Ltd., Shandong, China). It was extracted from corncob and contained 95% XOS with a degree of polymerization of from 2 to 7 and 5% xylose on the dry matter basis. The XOS was added in the diets at the expense of corn.

**TABLE 1:** Composition and nutrient levels of experimental diet (as-fed basis).

Ingredient	Content (%)^d^
Corn	62.78
Soybean meal (44% crude protein)	24.00
Fresh or oxidized fish oil^a^	2.00
Salt	0.30
Dicalcium phosphate	1.00
Calcium carbonate	9.34
DL-Methionine	0.12
Choline chloride (50%)	0.10
Premix^b^	0.33
Phytase	0.03
XOS95P^c^	±^e^
**Nutrient level**	
Metabolizable energy (MJ/kg)	11.64
Crude protein	16.5 (16.34)
Non-phytate phosphorus	0.33
Calcium	3.48
Lysine	0.79
Methionine	0.37
Methionine + cysteine	0.65
Threonine	0.60

*^*a*^Peroxide values of fresh and oxidized fish oil were 3.20 and 22.25 meq/kg, respectively.*

*^*b*^Premix supplied per kilogram of diet: vitamin A, 12,500 IU; vitamin D_3_, 4,125 IU; vitamin E, 15 IU; vitamin K_3_, 2 mg; thiamine, 1 mg; riboflavin, 8.5 mg; pyridoxine, 8 mg; vitamin B_12_, 0.04 mg; biotin, 0.1 mg; folic acid, 1.25 mg; Ca-pantothenate, 50 mg; niacin, 32.5 mg; Cu, 8 mg; Zn, 65 mg; Fe, 60 mg; Mn, 65 mg; Se, 0.3 mg; I, 1 mg.*

*^*c*^XOS95P, a mixture of 95% xylooligosaccharide (XOS) with the degree of polymerization 2–7 and 5% xylose on the dry matter basis.*

*^*d*^The value in parenthesis indicates analyzed value. Others are calculated values.*

*^*e*^XOS was supplemented at 200 and 400 mg/kg at the expense of corn.*

### Performance Measurement

Mortality was recorded as it occurred. Daily egg number, total egg weight, and biweekly feed consumption were recorded and calculated as hen-day egg production (EP), average egg weight (AEW), average daily feed intake (ADFI), feed conversion ratio (FCR) on a biweekly basis. EP, AEW, ADFI, and FCR were calculated for weeks 1–6, 7–12, and 1–12.

### Sample Collection

At the end of the feeding trial, one bird per replicate was randomly selected for sample collection. Blood samples were collected from wing vein, serum was isolated by centrifugation at 3,000×*g* for 10 min at 4°C and stored at −20°C for endotoxin analysis. After blood collection, these birds were slaughtered rapidly. Segments (approximately 2 cm in length) of the middle portion of the ileum (about 5 cm from Meckel’s diverticulum) were collected, washed with PBS, and fixed in 10% neutral-buffered formalin for histology. The remainder of ileum was removed, opened longitudinally, and gently rinsed with PBS. The mucosa was scraped aseptically by sterile glass slides, frozen as aliquots in liquid nitrogen, and stored at −80°C for sIgA determination and the quantification of gene expression. Additionally, cecal contents (approximately 1 g) were collected in sterile containers, and then frozen by immersion in liquid nitrogen, and stored at −80°C for intestinal microbiota analysis.

### Ileal Morphology Analysis

Samples were washed, dehydrated, clarified, and embedded in paraffin. Serial sections were cut at 5 μm thickness, placed on glass slides, deparaffinized in xylene, rehydrated, stained with hematoxylin and eosin, fixed with neutral balsam, and examined by light microscopy (BX51, Olympus Co., Tokyo, Japan). All reagents used were of analytical grade (Sinopharm Chemical Reagent Co., Ltd., Beijing, China). The morphometric indices evaluated were villus height (VH; from the tip of the villus to the villus–crypt junction), crypt depth (CD; from the base up to the crypt–villus transition region), and the villus height-to-crypt depth ratio (VCR) (Forte et al., 2016). The number of goblet cells (GCN) was counted on 100 columnar cells of villus mucosa at 400× magnification.

### Content of SIgA in the Ileal Mucosa

Ileal mucosa was homogenized in 10 volumes of ice-cold saline and centrifuged at 20,000×*g* for 10 min at 4°C. The supernatant was collected for sIgA determination using an assay kit (Jiancheng Bioengineering Institute, Nanjing, China). All the procedures were carried out according to the instructions of the manufacturer. The protein concentration was detected by the Coomassie Brilliant Blue method, using an assay kit (Jiancheng Bioengineering Institute, Nanjing, China), and the sIgA content was expressed as ng/g protein.

### Concentration of Endotoxin in Serum

Birds in the OFO/XOS_400_ group exhibited the greater ileal morphology and ileal mucosal sIgA content than those in the OFO/XOS_200_ group. Therefore, further determinations including serum endotoxin concentration, relative mRNA expression of tight junction proteins, and cecal microbial diversity and composition were only conducted among the FFO, OFO, and OFO/XOS_400_ groups to reveal the possible mechanism of the alleviation of XOS on intestinal mucosal impairment induced by OFO. Serum endotoxin concentration was measured using a chromogenic substrate assay kit (Jiancheng Bioengineering Institute, Nanjing, China). Briefly, 0.1 ml of serum was incubated with 0.1 ml of Limulus amebocyte lysate at 37°C for 45 min. After several subsequent reactions, the samples were read spectrophotometrically at 545 nm. The endotoxin level was calculated based on a standard curve of endotoxin concentration.

### Quantitative Real-Time PCR Analysis

Total RNA of the ileal mucosa was extracted using Trizol reagent (Tiangen Biotech Co., Ltd., Beijing, China) according to the manufacturer’s instructions. RNA concentration was determined using a NanoDrop 2000 spectrophotometer (Thermo Fisher Scientific, Waltham, MA), and the integrity was evaluated using agarose–ethidium bromide electrophoresis. Reverse transcription (RT) reactions were immediately performed using the FastQuant RT Kit (Tiangen Biotech Co., Ltd., Beijing, China). Real-time quantitative PCR was conducted in duplicate in the Bio-Rad C1000 thermal cycler (CFX-96 real-time PCR detection systems; Bio-Rad). PCR programs for all genes were as follows: 15 min at 95°C, 40 cycles of 95°C for 10 s, 60°C for 30 s. The relative gene expression levels were calculated using the 2^–ΔΔ*Ct*^ method (Livak and Schmittgen, 2001), and avian β-actin was used as reference gene. The primer sequences for the target genes [zonula occludens-2 (*ZO2*), claudin-1 (*CLDN1*), claudin-5 (*CLDN5*)] and β-actin are listed in Table 2.

**TABLE 2:** Primers used for quantitative real-time PCR.

Gene^a^	Primer sequences	Accession number	Length (bp)
*ZO2*	F:CGGCAGCTATCAGACCACTC	NM_204918	87
	R:CACAGACCAGCAAGCCTACAG		
*CLDN1*	F:CTGATTGCTTCCAACCAG	NM_001013611	140
	R:CAGGTCAAACAGAGGTACAAG		
*CLDN5*	F:CATCACTTCTCCTTCGTCAGC	NM_204201	111
	R:GCACAAAGATCTCCCAGGTC		
β*-actin*	F:ATGATATTGCTGCGCTCGTT	NM_205518.1	145
	R:TCTTTCTGGCCCATACCAACC		

*^*a*^ZO2, zona occludens-2; CLDN1, claudin-1; CLDN5, claudin-5.*

### DNA Extraction, 16S rRNA Amplification, and High-Throughput Sequencing

Total bacterial genomic DNA was extracted from cecal content samples using the E.Z.N.A Soil DNA Kit (Omega Bio-tek, Norcross, GA, United States) according to manufacturer’s instructions. The quality of DNA samples was checked with gel electrophoresis. Bacterial 16S rDNA sequences spanning the hypervariable regions v3–v4 were amplified using primers: 338F (5′-ACT CCT ACG GGA GGC AGC A-3′) and 806R (5′-GGA CTA CHV GGG TWT CTA AT-3′). The PCR reaction conditions were 2 min of denaturation at 95°C, followed by 25 cycles of 30 s at 95°C (denaturation), annealing at 55°C for 30 s, and extension at 72°C for 30 s, with a final extension of 5 min at 72°C. Amplicons were extracted from 2% agarose gels and purified using the AxyPrep DNA Gel Extraction Kit (Axygen Biosciences, Union City, CA, United States) according to the manufacturer’s instructions. Purified amplicons were qualified and sequenced using MiSeq platform with MiSeq Reagent Kit v3 at Shanghai Personal Biotechnology Co., Ltd. (Shanghai, China). The raw reads were deposited into the NCBI Sequence Read Archive (SRA) database (Accession Number: PRJNA673331). The raw pair-end sequences from the original DNA fragments were demultiplexed and quality-filtered using the Quantitative Insights Into Microbial Ecology (QIIME, version 1.17). Only sequences that overlap by more than 10 bp were assembled according to their overlap sequence. Operational taxonomic units (OTUs) were clustered with 97% sequence identity using UPARSE (version 7.1), and the chimera sequences were identified and removed to obtain effective tags using UCHIME. Shannon and Simpson diversity indices, and Ace and Chao richness estimators were included in the alpha diversity analysis by using the MOTHUR v1.31.2. Principal coordinate analysis (PCoA) and partial least squares discriminant analysis (PLS-DA) based on unweighted UniFrac distance were conducted to compare the bacterial community structures across all samples and to establish beta diversity. The significance of differentiation of microbial structure among groups was statistically tested by analysis of similarity (ANOSIM). Statistical tests for differentially abundant taxa were performed using the linear discriminant analysis (LDA) effect size (LEfSe) method with an alpha value of 0.05 for the Kruskal–Wallis test among classes, and the threshold for the log_1__0_LDA score was set as 2.0. Metagenome functional content from 16S rDNA was predicted using PICRUSt (Langille et al., 2013), based on the Clusters of Orthologous Groups (COG) and Kyoto Encyclopedia Genes and Genomes (KEGG) databases. Pearson correlation analysis was performed for the correlations between phylotypes and phenotypes.

### Statistical Analysis

Differences in performance, ileal morphology, ileal mucosal sIgA, serum endotoxin, relative expression of tight junction proteins, and alpha diversity indices of cecal microbiota were analyzed using one-way ANOVA followed by Duncan’s multiple range test (SPSS, version 19.0, Chicago, IL). Wilcoxon rank-sum test was employed to explore the differences in cecal microbiota in terms of the relative abundances of species between groups FFO and OFO, and between groups OFO and OFO/XOS_400_. Significance was set at *P* < 0.05, and 0.05 < *P* < 0.10 was considered as a trend toward significance. Data are expressed as the means and pooled SEM.

## Results

### Performance

No birds died during entire experimental period. Birds in the OFO group presented a lower (*P* < 0.05) egg production from weeks 7 to 12 of the experiment, which was concomitant with poor (*P* < 0.10) FCR from weeks 7 to 12 and 1 to 12 than those in the FFO group (Table 3). However, there were no significant differences in EP and FCR among the FFO, OFO/XOS_200_, and OFO/XOS_400_ groups from weeks 7 to 12 or 1 to 12 (*P* > 0.05). Affected by the temperature of layer house, egg production of all birds in the first 6 weeks was lower than that in the second 6 weeks.

**TABLE 3:** Effect of dietary xylooligosaccharide supplementation on performance of laying hens fed an oxidized fish oil diet^1^.

Item^2^	Experimental treatment^3^				SEM	*P*-value
	FFO	OFO	OFO/XOS_200_	OFO/XOS_400_		
**Weeks 1–6**						
EP (%)	88.00	87.67	87.17	86.14	0.75	0.849
AEW (g)	63.25	63.10	63.24	63.81	0.36	0.915
ADFI (g)	114.93	114.17	112.26	113.59	0.72	0.625
FCR	2.01	2.01	1.98	2.00	0.02	0.932
**Weeks 7–12**						
EP (%)	93.78^a^	88.34^b^	91.08^ab^	90.58^ab^	0.61	0.011
AEW (g)	65.60	65.18	65.45	65.76	0.34	0.949
ADFI (g)	120.34	121.15	119.62	119.20	0.45	0.445
FCR	1.99	2.11	2.02	2.04	0.02	0.051
**Weeks 1–12**						
EP (%)	90.80	87.73	89.03	88.08	0.51	0.139
AEW (g)	64.42	64.14	64.34	64.78	0.34	0.935
ADFI (g)	117.63	117.96	115.94	116.40	0.51	0.553
FCR	1.96	2.05	2.00	2.03	0.01	0.066

*^1^n = 8 replicates per treatment.*

*^2^EP, egg production; AEW, average egg weight; ADFI, average daily feed intake; FCR, feed conversion ratio (feed/egg; g/g).*

*^3^FFO, fresh fish oil diet; OFO, oxidized fish oil diet; OFO/XOS_200_, oxidized fish oil diet + 200 mg/kg xylooligosaccharide; OFO/XOS_400_, oxidized fish oil diet + 400 mg/kg xylooligosaccharide.*

*^*a,b*^Within a row, means with no common small letters (a–c) differ significantly (P < 0.05).*

### Ileal Morphology

The ileal morphology of laying hens fed the experimental diets is shown in Table 4 and Supplementary Figure S1. No significant differences were detected in ileal VH, CD, the VCR, or GCN between FFO and OFO groups (*P* > 0.05). However, hens in the OFO/XOS_400_ group showed higher VH, VCR, and GCN in the ileum compared with those in the other groups (*P* < 0.05).

**TABLE 4:** Effect of dietary xylooligosaccharide supplementation on ileal morphology of laying hens fed an oxidized fish oil diet^1^.

Item^2^	Experimental treatment^3^				SEM	*P-*value
	FFO	OFO	OFO/XOS_200_	OFO/XOS_400_		
VH (μm)	611.85^b^	594.94^b^	613.40^b^	749.70^a^	19.05	0.007
CD (μm)	159.28	164.75	161.19	165.09	4.32	0.691
VCR	3.99^b^	3.64^b^	3.82^b^	4.59^a^	0.10	0.002
GCN	21.13^b^	19.33^b^	20.23^b^	24.91^a^	0.55	<0.001

*^1^n = 8 replicates per treatment.*

*^2^VH, villus height; CD, crypt depth; VCR, the villus height to crypt depth ratio; GCN, goblet cells number.*

*^3^FFO, fresh fish oil diet; OFO, oxidized fish oil diet; OFO/XOS_200_, oxidized fish oil diet + 200 mg/kg xylooligosaccharide; OFO/XOS_400_, oxidized fish oil diet + 400 mg/kg xylooligosaccharide.*

*^*a,b*^Within a row, means with no common small letter differ significantly (P < 0.05).*

### Concentrations of SIgA in the Ileal Mucosa and the Endotoxin in Serum

**FIGURE 1:**
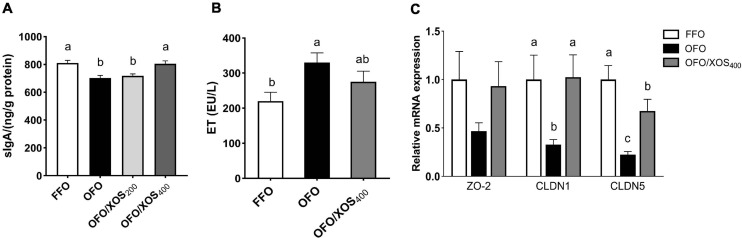
Effect of dietary xylooligosaccharide supplementation on ileal barrier of laying hens fed oxidized fish oil. **(A,B)** Show the secretory immunoglobulin A (sIgA) content of ileal mucosal and the endotoxin concentration in serum, respectively. **(C)** Shows the gene expressions of zona occludens-2 (*ZO2*), claudin-1 (*CLDN1*), and claudin-5 (*CLDN5*) in the ileal mucosa. Data are presented as the means ± *SEM* (*n* = 8). FFO, fresh fish oil diet; OFO, oxidized fish oil diet; OFO/XOS_200_, oxidized fish oil diet + 200 mg/kg of xylooligosaccharide; OFO/XOS_400_, oxidized fish oil diet + 400 mg/kg of xylooligosaccharide. Bars with no common letter indicate statistical differences among treatments (*P* < 0.05).

The content of sIgA in the ileal mucosa of laying hens at week 12 is shown in Figure 1A. There was a significant decrease (*P* < 0.05) in ileal mucosal sIgA content of hens in the OFO and OFO/XOS_200_ groups, compared with the FFO group; however, it was higher (*P* < 0.05) in the OFO/XOS_400_ group than in the OFO group.

The increased (*P* < 0.05) serum endotoxin concentration induced by the oxidized fish oil diet was diminished by 400 mg/kg of XOS supplementation, reflected by no significant difference between FFO and OFO/XOS_400_ groups (*P* > 0.05) (Figure 1B).

### Relative mRNA Expression of Tight Junction Proteins

There were decreases (*P* < 0.05) in the relative expression of ileal mucosal *CLDN1* and *CLDN5* of the layers in the OFO group, compared with the FFO group (Figure 1C); however, they were both increased (*P* < 0.05) in the layers of the OFO/XOS_400_ group when compared with those in the OFO group.

### Diversity of Gut Microbiota

**FIGURE 2:**
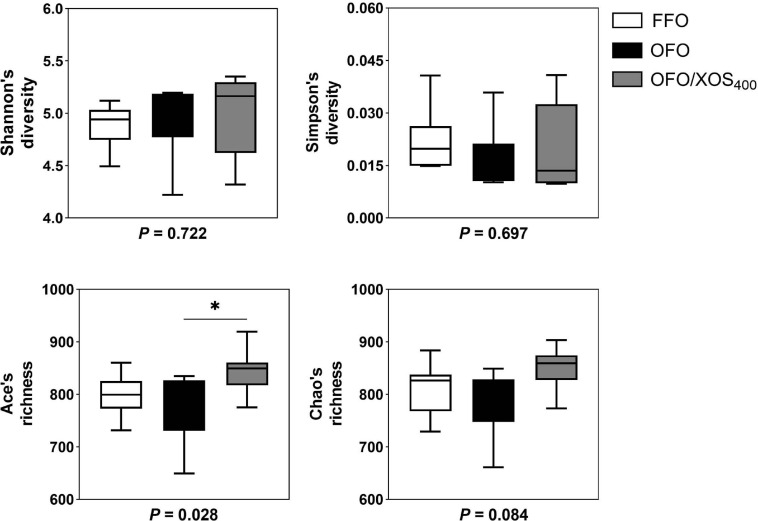
Influence of xylooligosaccharide on cecal bacterial alpha diversity of laying hens fed oxidized fish oil. Four different alpha diversity metrics, Shannon and Simpson diversity indices, and Ace and Chao richness estimators, were compared among three groups. FFO, fresh fish oil diet; OFO, oxidized fish oil diet; OFO/XOS_400_, oxidized fish oil diet + 400 mg/kg xylooligosaccharide. *P*-values given below each boxplot were estimated by Duncan’s multiple range test. ^∗^ means *P* < 0.05.

**FIGURE 3:**
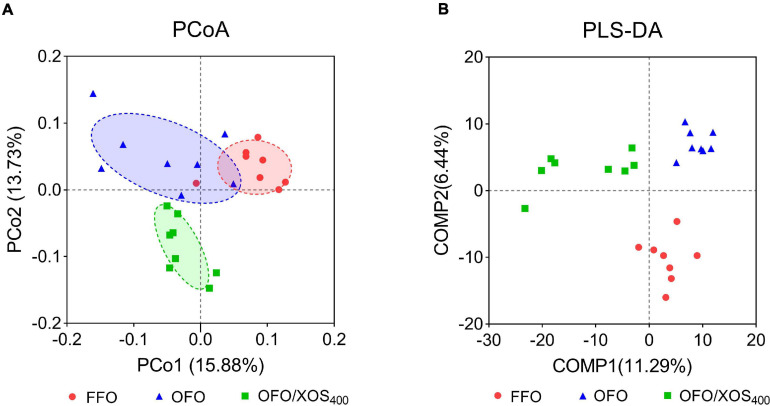
Beta-diversity analysis of cecal microbiota among three groups. **(A)** Principal coordinate analysis (PCoA) based on unweighted UniFrac distance calculated from OTU abundance matrix. The horizontal axis represents the first principal coordinate, and the vertical axis means the second one. **(B)** Partial least squares discriminant analysis (PLS-DA). The horizontal axis represents the first component of PLS-DA, and the vertical axis means the second one. The percentages in parentheses represent the explanatory values of the principal coordinates or the components for the difference in sample composition. FFO (red circles), fresh fish oil diet; OFO (blue triangles), oxidized fish oil diet; OFO/XOS_400_ (green rectangles), oxidized fish oil diet + 400 mg/kg xylooligosaccharide.

Bacterial alpha diversity in cecal microbiota was estimated using Shannon, Simpson, Ace, and Chao indices of diversity and richness (Figure 2). No significant differences in Shannon or Simpson indices were found among the 3 groups (*P* > 0.05). Supplemental XOS (400 mg/kg) increased Ace (*P* = 0.028) and Chao (*P* = 0.084) indices of the cecal microbiota in laying hens fed oxidized fish oil. PCoA based on unweighted UniFrac distance revealed a separation of microbial communities among the 3 groups (PCo1, 15.88%; PCo2, 13.73%) (Figure 3A). PLS-DA plot also defined groups where samples from different groups occupied distinct positions (COMP1, 11.29%; COMP2, 6.44%) (Figure 3B). Results were supported by statistics obtained from ANOSIM analysis (Supplementary Table S1).

### Composition of Gut Microbiota

**FIGURE 4:**
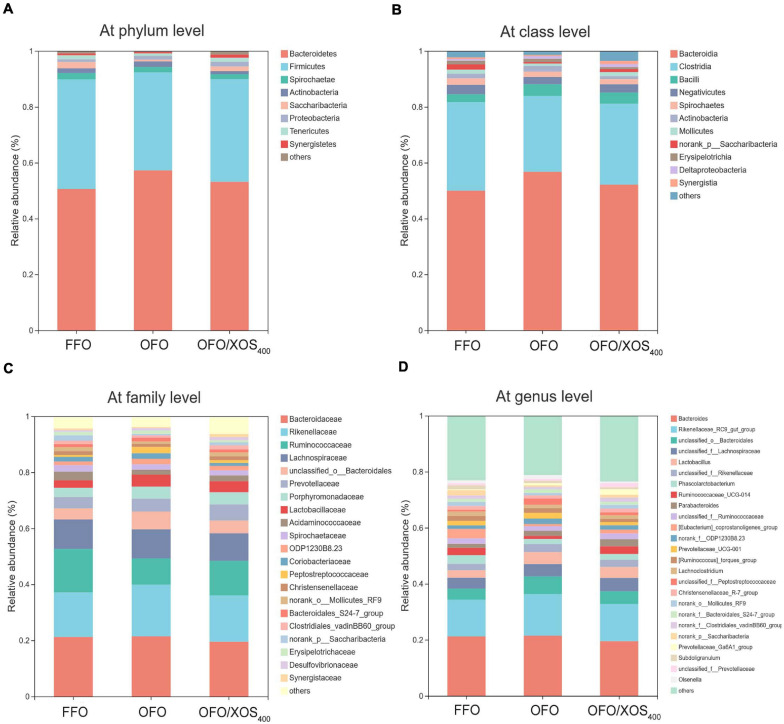
Effect of dietary xylooligosaccharide supplementation on cecal microbial composition at different taxonomic levels of laying hens fed oxidized fish oil **(A)** at phylum level, **(B)** at class level, **(C)** at family level, and **(D)** at genus level. Those abundance below 1% were classified as “others.” The relative abundance value shown in the plot represent the average of eight samples in each group. FFO, fresh fish oil diet; OFO, oxidized fish oil diet; OFO/XOS_400_, oxidized fish oil diet + 400 mg/kg xylooligosaccharide.

Taxonomic compositions of the cecal microbiota were analyzed at the different levels using the RDA classifier. The dominant phyla across all the groups were *Bacteroidetes* and *Firmicutes*, which together contributed greater than 88% of the whole phyla (Figure 4A). A higher abundance of *Bacteroidetes* with a lower abundance of *Firmicutes* were observed in layers fed OFO compared to those in the FFO group. However, XOS addition partially reversed these changes found in the OFO group. Within *Firmicutes*, the majority belonged to the classes *Clostridia*, *Bacilli*, and *Negativicutes* (Figure 4B). At the family level, the cecal microbiota of layers was dominated by *Bacteroidaceae*, *Rikenellaceae*, *Ruminococcaceae*, and *Lachnospiraceae* (Figure 4C). OFO treatment led to a reduced abundance of *Ruminococcaceae* with an increased abundance of *Rikenellaceae* compared with the FFO group, whereas dietary XOS supplementation increased the abundance of *Ruminococcaceae* to some extent. Genus level analysis showed that the *Bacteroides* and *Rikenellaceae_RC9_gut_group* accounted for the largest proportion of the community (Figure 4D). The abundance of *Rikenellaceae_RC9_gut_group* was increased in layers fed OFO but diminished with XOS addition.

**FIGURE 5:**
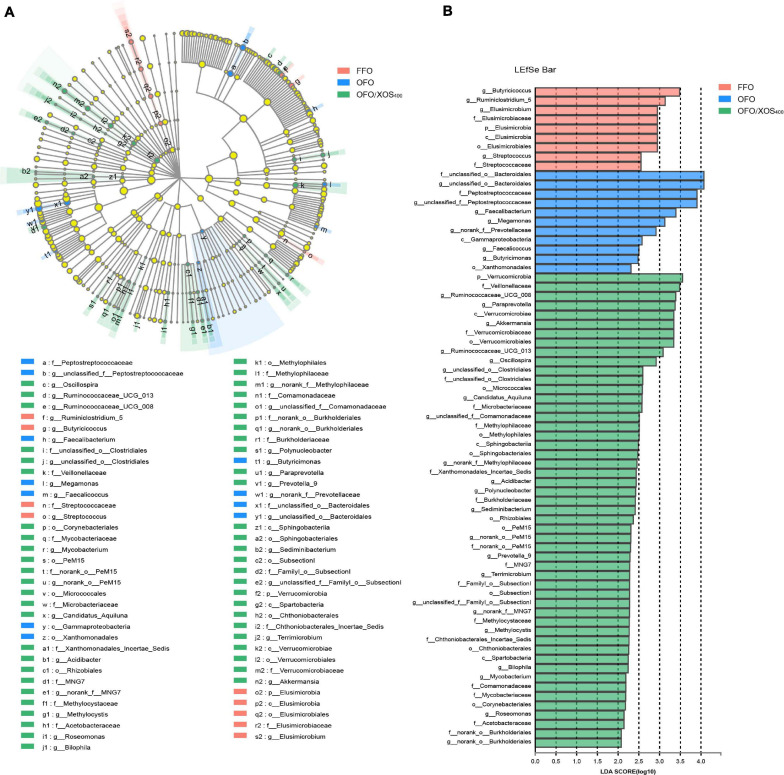
LEfSe identified the most differentially abundant taxa enriched in cecal microbiota among groups FFO (fresh fish oil diet), OFO (oxidized fish oil diet), and OFO/XOS_400_ (oxidized fish oil diet + 400 mg/kg xylooligosaccharide). **(A)** Cladogram generated from LEfSe analysis, where red, blue, and green circles represent taxa of greater abundance of hens in the FFO, OFO, and OFO/XOS_400_ groups, respectively. Yellow circles mean non-significant differences. The diameters of the circles are proportional to the taxon’s abundance. **(B)** Histogram of the LDA scores computed for features differentially abundant among FFO (red bars), OFO (blue bars), and OFO/XOS_400_ (green bars) groups. Species with significant difference that have an LDA score greater than 2.0 are presented. The length of the histogram represents the LDA score, which can be interpreted as the effect size of each differentially abundant feature.

LEfSe analysis was applied to identify the significant differentially abundant OTUs for the entire microbiota at levels from phylum to genus (LDA > 2.0). As shown in Figure 5, the cecal microbiota in the FFO group was enriched with *Streptococcaceae* (*Streptococcus*), *Butyricicoccus*, and *Ruminiclostridium_5* as well as *Elusimicrobia* and its derivatives (*Elusimicrobia*, *Elusimicrobiales*, *Elusimicrobiaceae*, and *Elusimicrobium*). Birds in the OFO group exhibited increased abundances of family *Peptostreptococcaceae* and genera *Faecalibacterium*, *Butyricimonas*, and *Faecalicoccus*. In the OFO/XOS_400_ group, LEfSe highlights substantial bacterial members enriched in the cecum, some of which accounted for greater proportions and had higher LDA scores such as phylum *Verrucomicrobia* and its derivatives (*Verrucomicrobiae*, *Verrucomicrobiales*, *Verrucomicrobiaceae*, and *Akkermansia*) in addition to the genera *Paraprevotella*, *Prevotella_9*, and *Oscillospira*. The Wilcoxon rank-sum test was further performed to reveal the differences in the microbial composition (Table 5). Microbiota in the cecum of birds fed OFO presented lower (*P* < 0.05) abundances of *Verrucomicrobia* (*Akkermansia*), *Elusimicrobia* (*Elusimicrobium*), and *Ruminococcaceae_UCG-008* when compared with those in the FFO group. Besides, OFO treatment triggered a higher abundance of *Fusobacteria* (*P* < 0.05) than those in FFO group. There were elevations (*P* < 0.05) in the abundances of *Verrucomicrobia* (*Verrucomicrobiaceae*, *Akkermansia*), *Paraprevotella*, *Oscillospira*, and *Ruminococcaceae_UCG-013* following XOS addition, which also decreased (*P* < 0.05) the abundance of *Erysipelatoclostridium* compared with that in the OFO group.

**TABLE 5:** Differences in relative abundance (%) of cecal microbiota at phylum and genus in laying hens in different groups^1^.

Taxonomic group^2^	Experimental treatment^3^			*P*-value	
	FFO	OFO	OFO/XOS_400_	FFO vs. OFO	OFO vs. OFO/XOS_400_
**Phylum**					
*Verrucomicrobia*	0.439 ± 0.250	0.096 ± 0.064	0.726 ± 0.498	0.024	0.046
*Elusimicrobia*	0.220 ± 0.124	0.057 ± 0.035	0.115 ± 0.078	0.002	0.155
*Fusobacteria*	0.003 ± 0.003	0.027 ± 0.025	0.004 ± 0.003	0.046	0.188
**Genus**					
*Akkermansia*	0.028 ± 0.014	0.001 ± 0.001	0.383 ± 0.152	0.018	0.013
*Paraprevotella*	0.437 ± 0.470	0.223 ± 0.248	0.666 ± 0.349	0.103	0.018
*Oscillospira*	0.105 ± 0.084	0.040 ± 0.044	0.186 ± 0.104	0.058	0.003
*Erysipelatoclostridium*	0.162 ± 0.114	0.356 ± 0.220	0.143 ± 0.082	0.074	0.027
*Elusimicrobium*	0.220 ± 0.124	0.057 ± 0.035	0.078 ± 0.055	0.002	0.481
*Butyricimonas*	0.093 ± 0.052	0.135 ± 0.057	0.082 ± 0.031	0.045	0.027
*R. UCG-008*	0.393 ± 0.239	0.159 ± 0.184	0.605 ± 0.690	0.031	0.052
*R. UCG-013*	0.352 ± 0.172	0.222 ± 0.134	0.451 ± 0.300	0.141	0.014
*P. unclassified*	0.668 ± 0.797	2.246 ± 1.213	0.960 ± 0.423	0.014	0.052

*^1^Data are presented as the means ± standard deviation (n = 8).*

*^2^R. UCG-008, Ruminococcaceae_UCG-008; R. UCG-013, Ruminococcaceae_UCG-013; P. unclassified, Peptostreptococcaceae unclassified.*

*^3^FFO, fresh fish oil diet; OFO, oxidized fish oil diet; OFO/XOS_400_, oxidized fish oil diet + 400 mg/kg xylooligosaccharide.*

### Functional Prediction of Gut Microbiota

Alterations in the presumptive functions of the cecal microbiota of layers were evaluated by PICRUSt analysis. Metagenomic prediction based on COG categories (Supplementary Figure S2A) revealed that the functional pathways enriched within the microbiota were those corresponding to carbohydrate transport and metabolism, amino acid transport and metabolism, general function prediction only, transcription, replication, recombination and repair, cell wall/membrane/envelope biogenesis, as well as translation, ribosomal structure, and biogenesis. In terms of the KEGG prediction (Supplementary Figure S2B), the microbiota represented in the cecum were mainly associated with the following biological processes: membrane transport, carbohydrate metabolism, amino acid metabolism, replication and repair, energy metabolism, and translation. However, there were no differences (*P* > 0.10) noted in the predicted pathways of the microbiota among all groups.

### Correlation Between Phylotypes and Phenotypes

**FIGURE 6:**
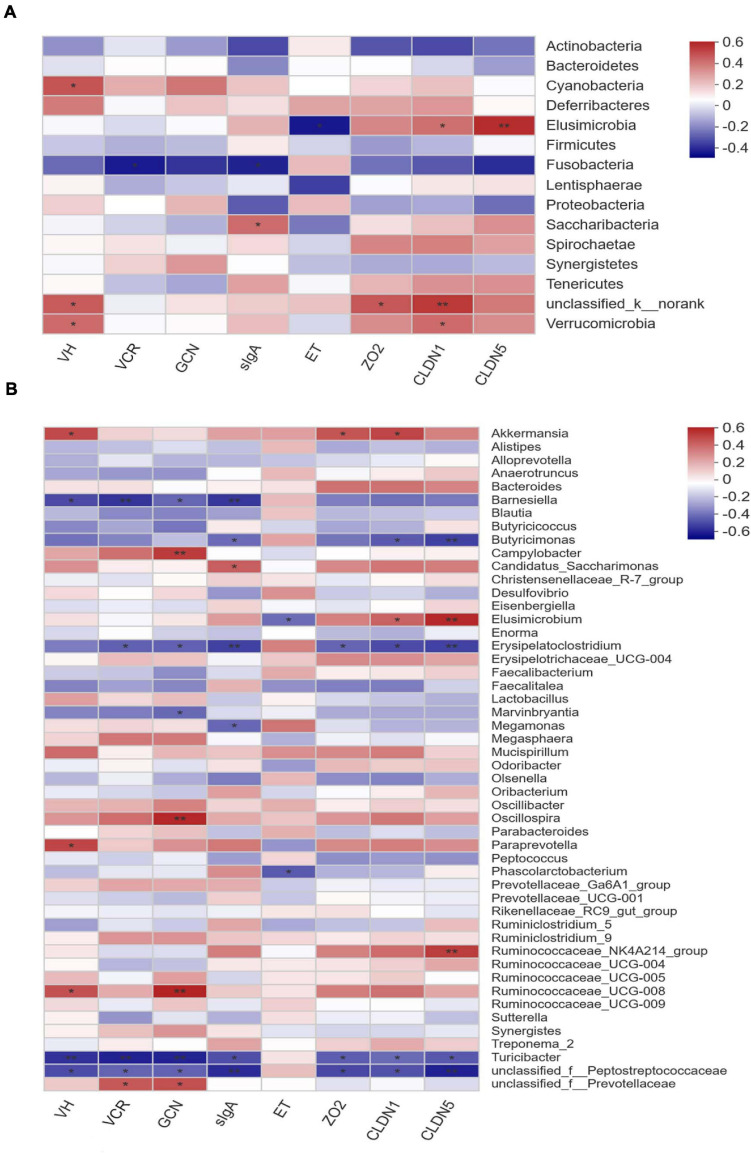
Correlation matrix between key phylotypes (**A** at phylum level, **B** at genus level) and phenotypes in laying hens. VH, villus height; VCR, the villus height-to-crypt depth ratio; GCN, goblet cell number; sIgA, secretory immunoglobulin A; ET, endotoxin; *ZO2*, zona occludens-2; *CLDN1*, claudin-1; *CLDN5*, claudin-5. The depth of colors ranging from blue to red represents the magnitude of correlation. The OTUs were organized according to their Pearson correlation coefficient. Significant correlations are noted by *0.01 < *P* ≤ 0.05, **0.001 < *P* ≤ 0.01.

Pearson correlation analysis was conducted to estimate the association between phylotypes in cecal microbiota and phenotypes of layers. As shown in Figure 6A, the abundance of phylum *Verrucomicrobia* was positively correlated (*P* < 0.05) with ileal VH and the expression of *CLDN1* in ileal mucosa. In contrast, the abundance of phylum *Fusobacteria* showed negative correlation (*P* < 0.05) with the VCR of ileum and sIgA content in ileal mucosa. Comparatively, *Elusimicrobia* was positively correlated with the expression of *CLDN1* (*P* < 0.05) and *CLDN5* (*P* < 0.01), but negatively correlated (*P* < 0.05) with the endotoxin concentration in serum. In terms of the cecal microbiota at genus level, the abundances of *Erysipelatoclostridium*, *Turicibacter*, and an unclassified member of *Peptostreptococcaceae* were negatively correlated (*P* < 0.05) with ileal VH, the VCR, GCN, and sIgA concentration, as well as the expression of ileal mucosal *ZO2*, *CLDAN1*, and *CLDN5* (Figure 6B). Conversely, the abundances of *Oscillospira*, *Paraprevotella*, and *R. UCG-008* were positively correlated (*P* < 0.05) with ileal VH and/or GCN. In addition, the relative abundance of *Akkermansia* was also positively associated (*P* < 0.05) with ileal VH and the expression of *ZO2* and *CLDN1*, whereas negative correlations (*P* < 0.05) were detected between *Butyricimonas* and ileal mucosal expression of *CLDN1*, *CLDN5*, as well as sIgA concentration. In contrast, the abundance of *Elusimicrobium* was positively correlated with ileal mucosal *CLDN1* (*P* < 0.05) and *CLDN5* (*P* < 0.01) expression, but negatively associated (*P* < 0.05) with the endotoxin content in serum.

## Discussion

Previous studies evidenced that supplemental XOS was associated with an improvement on performance of poultry (Min et al., 2016; Ribeiro et al., 2018; Craig et al., 2020). Similarly, poor performance of layers induced by OFO treatment in the current study was relieved following XOS addition, reflected by the improved EP and FCR. Reduced performance in the OFO group may be caused by some secondary lipid peroxidation products in oxidized fish oil, such as α and β-unsaturated hydroxyaldehydes (Tan et al., 2019). These toxicants are highly toxic and readily absorbed, which can damage the intestinal proliferative cells and functional cells of birds (Dibner et al., 1996). Intestinal morphology is considered to be relevant to animal performance because an expanded absorptive surface area means greater digestion and absorption (Montagne et al., 2003; Yamauchi, 2007). In concert with previous studies (De Maesschalck et al., 2015; Min et al., 2016; Ding et al., 2018), the current study showed that hens fed OFO diet with XOS supplementation (400 mg/kg) exhibited higher ileal VH and VCR, which might be due to its role in stimulating butyrate-producing microbes to produce butyrate to fuel intestinal epithelial cells (Liu et al., 2018). In order to elucidate the potential mechanism underlying the improved EP and FCR and improved ileal morphology following XOS addition, the variation of ileal mucosal barrier was further investigated.

The intestinal mucosal barrier is the first innate line of defense against pathogen invasion via the cooperation of epithelial cells, mucus layers, sIgA, and tight junctions (Derrien et al., 2017; Ma et al., 2018; Xie et al., 2019b). Goblet cells are differentiated epithelial cells that play a vital role in intestinal mucosal barrier maintenance via the secretion of mucus (Knoop and Newberry, 2018). In the current study, supplemental XOS (400 mg/kg) led to an increased GCN in the ileum of layers, suggesting a possible reinforcement of mucus shield that could be helpful in protecting against intestinal mucosal injury. SIgA secreted by epithelial cells is the principal immunoglobulin in the intestinal mucosal mucus, which was viewed as a key indicator of intestinal immune system (Belkaid and Hand, 2014; Azzam et al., 2017). Similar to a previous study (Min et al., 2016), our results showed that the reduced sIgA content in ileal mucosa induced by OFO was reversed by XOS supplementation at 400 mg/kg, implying an improvement of intestinal immune function in laying hens. Furthermore, tight junction proteins are intercellular junctional molecules that could control intestinal permeability thus thwarting the entry of pathogen and toxins (Barekatain et al., 2019). Therefore, reductions in mRNA expression of *CLDN1* and *CLDN5* in ileal mucosa observed in birds fed OFO provided a clue that intestinal mucosal barrier was impaired by OFO treatment. In support of this view, hens receiving the OFO diet without XOS addition presented a higher serum endotoxin level which served as an indicator of intestinal barrier damage (Mani et al., 2012). This increase in the concentration of serum endotoxin in the OFO group might be due to the lipid hydroperoxides generated from the oxidation of fish oil, which could induce a loss of membrane integrity, dysregulation of membrane transport, and alteration in membrane permeability (Wijeratne and Cuppett, 2006; Dasilva et al., 2017). However, in the current study, XOS addition (400 mg/kg) resulted in upregulated ileal mucosal gene expression of *CLDN1* and *CLDN5* along with reduced serum endotoxin level of layers fed OFO diet, bringing an evidence for the alleviation of OFO-induced intestinal mucosal impairment of laying hens. Similar results were also described by some previous studies, in which dietary XOS supplementation protected intestinal mucosal barrier against challenge in poultry (Eeckhaut et al., 2008; Pourabedin et al., 2017).

The cecum of poultry contains the most detailed information regarding gut microbiota and is the key region for bacterial fermentation of oligosaccharides (Pourabedin and Zhao, 2015). Given the essential roles of cecal microbiota in mediating the manipulation of intestinal barrier by dietary intervention (Wang et al., 2019a; Miao et al., 2020), the alteration of cecal microbiota in the current study was further analyzed. The high species diversity reflects a more stable microbiota community and prevents the colonization of pathogens, thus benefiting to productivity of the host bird (Janczyk et al., 2009; Han et al., 2017). Herein, results of alpha diversity analysis showed that XOS addition (400 mg/kg) increased cecal microbial richness in layers fed OFO diet, which was consistent with previous studies (Ou et al., 2016; Han et al., 2020). In addition, data from analysis of beta diversity showed significant clustering according to experimental groups, demonstrating that cecal microbial community structure was both affected by OFO diets with and without XOS addition. Notably, samples in the FFO and OFO/XOS_400_ groups were also clustered separately (Supplementary Table S1). We thus speculated that XOS addition modulated cecal microbiota of layers and not through restoring the bacterial structure that was disturbed by OFO treatment. Changes in microbial composition and some specific taxon in the current study were further analyzed.

Data showed that the cecal microbial composition of birds fed OFO was shifted at the phylum level by favoring *Bacteroidetes* at the expense of *Firmicutes*, whereas XOS addition increased the abundance of *Firmicutes* and reduced the abundance of *Bacteroidetes*, which is consistent with our previous study (Zhou et al., 2020). The abundance of *Firmicutes* has been proven to be positively correlated with energy and nutrient absorption, whereas an increase in fecal *Bacteroidetes* is associated with poor nutrient digestibility (Turnbaugh et al., 2006; Jumpertz et al., 2011). Therefore, the increased abundance of *Firmicutes* and the reduced abundance of *Bacteroidetes* in cecum may contribute to the melioration of OFO-induced poor FCR of layers following XOS addition. Additionally, XOS addition reversed a reduction in the abundance of *Ruminococcaceae* and an increase in the abundance of *Rikenellaceae* (*Rikenellaceae_RC9_gut_group*) caused by OFO treatment. As a member of SCFA producers, *Ruminococcaceae* has been illustrated to be responsible for the degradation of various polysaccharides and fibers (Hooda et al., 2012), leading to a positive correlation between *Ruminococcaceae* and feed efficiency in chickens (Torok et al., 2011). Besides, it was also reported that the abundance of *Ruminococcaceae* significantly and inversely correlated with intestinal permeability, thus benefiting gut barrier function (Leclercq et al., 2014). *Rikenellaceae* has been referred as indicators of inflammation that can recognize endotoxemia markers in animals (Zhai et al., 2017; Wang et al., 2018). Therefore, the altered abundance of *Rikenellaceae* among groups in the present study might be related to the different serum endotoxin content.

Excluding the dominant bacteria, some other representative species were identified as biomarkers to distinguish cecal microbiota of layers among groups. In the OFO group, LefSe highlights the greater differential abundances of family *Peptostreptococcaceae*, and genera *Faecalibacterium*, *Butyricimonas*, and *Faecalicoccus*. *Peptostreptococcaceae* in the human gut represented a maleficent bacterial group closely related to fatty liver and ulcerative colitis (Jiang et al., 2015; Lavelle et al., 2015). *Faecalibacterium* is a well-known colonizer in the cecum of poultry that participated in the intestinal proinflammatory response stimulated by recycled poultry litter (Shanmugasundaram et al., 2011; Wang et al., 2016). *Butyricimonas* and *Faecalicoccus* were rarely mentioned in poultry, as they were recently described and classified as novel genera (Kanno et al., 2015; Forbes et al., 2018). Nevertheless, *Butyricimonas* exhibited negative association with intestinal mucosal sIgA content and expression of *CLDN1* and *CLDN5* in the current study. The Wilcoxon rank-sum test further revealed some differential species between OFO and FFO treatments. For example, OFO treatment triggered a significantly increased *Fusobacteria*, which possesses virulence characteristics that can adhere and invade into host epithelial cells to elicit proinflammatory response (Strauss et al., 2011; Kostic et al., 2012). Consistently, the negative correlations between *Fusobacteria* and ileal VCR and sIgA content were observed in the current study. These increased potential pathogens mentioned above might contribute to the impairment of intestinal mucosal barrier induced by OFO treatment. In addition, *Akkermansia* is a strict anaerobe and uses the mucin in gut mucus as the sole source of carbon and nitrogen elements (Derrien et al., 2017). Some members of *Elusimicrobia* were characterized by small size with thin and fragile outer envelope, making them sensitive to adverse conditions (Zheng et al., 2015; Martinez-Fernandez et al., 2019). Thus, the reductions of *Akkermansia* and *Elusimicrobia* (*Elusimicrobium*) in the OFO group might be the consequences of the impaired intestinal mucosal barrier, including the possible reduction of mucus generation. Taken together, OFO diet exactly caused a dysbiosis in cecal microbiota of laying hens, which was probably involved in the impairment of intestinal mucosal barrier though it was hard to determine which came first, the microbiota dysbiosis or an intestinal mucosal damage.

Despite the fact that it is hard to distinguish whether dysbiosis of the cecal microbiota is the cause or consequence of intestinal mucosa damage, many salutary effects of microbiota modulation on gut mucosal barrier by dietary intervention have been already proven in poultry (Pourabedin et al., 2017; Wang et al., 2019b). As a kind of typical prebiotic, XOS has been reported to be beneficial to the health and performance of birds through modulating the gut microbiota (De Maesschalck et al., 2015; Ding et al., 2018). Herein, XOS addition (400 mg/kg) enriched the abundances of *Verrucomicrobia* (*Akkermansia*), *Paraprevotella*, *Prevotella_9*, and *Oscillospira* in the cecum of layers. A previous publication classified that some members of *Verrucomicrobia* were performance-linked species in cecum of chickens (Torok et al., 2011), of which *Akkermansia* can be served as the first and the most representative one (Zhang et al., 2019). It was reported that *Akkermansia* spp. not only participate in mucosal immune regulation but also improve the integrity of the intestinal epithelial cells and the thickness of the mucus layer, thus enhancing intestinal mucosal barrier (Everard et al., 2013; Reunanen et al., 2015). Consistently, our results showed that abundance of *Akkermansia* was positively associated with ileal VH and the expression of *ZO2* and *CLDN1*. *Paraprevotella* and *Prevotella* enriched by XOS treatment were also described by a recent research (Fei et al., 2020), in which they were associated with butyrate generation in the gut because of their capability to degrade cellulose and polysaccharides, which was favorable to FCR of animals fed plant-based diets (Gorvitovskaia et al., 2016; Shah et al., 2018). Besides, *Prevotella* abundance was also associated with a higher production of luminal sIgA, implying its role in intestinal immune regulation (Mach et al., 2015). Similarly, *Oscillospira*, a predominant genus in chicken cecal microbiota (Wang et al., 2016), was proved to be negatively correlated with intestinal inflammatory diseases (Gophna et al., 2017). Results of Wilcoxon rank-sum test between OFO and OFO/XOS_400_ groups basically support the alterations of cecal microbiota revealed by LefSe analysis. Furthermore, a significant reduction of the abundance of *Erysipelatoclostridium* was observed in OFO/XOS_400_ group compared to that in the OFO group. *Erysipelatoclostridium* was considered as an opportunistic pathogen (Shao et al., 2017) that exhibited negative associations with ileal VH, VCR, ileal mucosal sIgA content, and expression of *ZO2*, *CLDN1*, and *CLDN5* in the current study. To sum up, alterations of these bacterial phylotypes suggested that the cecal microbiota of layers fed with XOS became more favorable in protecting intestinal mucosal barrier from impairments by OFO and further improving production performance. Interestingly, only little bacteria altered by OFO treatment was reversed following XOS addition (400 mg/kg) in layers, which supports our hypothesis that XOS addition regulated OFO-induced gut microbiota dysbiosis through remodeling rather than restoring the microbial composition. Further studies are needed to elucidate the potential mechanism.

## Conclusion

Supplemental XOS attenuated OFO-induced impairment in production performance and fortified ileal morphology by alleviating intestinal mucosal barrier dysfunction in laying hens, which could be associated to the increase in microbial richness and remodeling of microbial composition. The current study would expand our knowledge concerning the roles of gut microbiota in mediating the manipulation of intestinal mucosal barrier by dietary intervention and can help to improve the efficiency in applying XOS in chicken production.

## Data Availability Statement

The datasets presented in this study can be found in online repositories. The names of the repository/repositories and accession number(s) can be found in the article/Supplementary Material.

## Ethics Statement

The animal study was reviewed and approved by the Animal Care and Use Committee of the Feed Research Institute of the Chinese Academy of Agricultural Sciences (ACE-CAAS- 20180915).

## Author Contributions

JW and JZ conceived and designed the experiments. JZ performed animal experiments, analyzed the data, and wrote the manuscript. JW assisted with data analysis and manuscript writing. GQ, KQ, SW, YF, and HZ supervised and provided continuous guidance for the experiment. All authors have discussed the results and reviewed the manuscript, read, and approved the final manuscript.

## Conflict of Interest

The authors declare that the research was conducted in the absence of any commercial or financial relationships that could be construed as a potential conflict of interest.

## Abbreviations

XOS: xylooligosaccharide
OFO: oxidized fish oil
FFO: fresh fish oil
SIgA: secretory immunoglobulin A
VH: villus height
CD: crypt depth
VCR: the villus height to crypt depth ratio
*ZO2*: zonula occludens-2
*CLDN1*: claudin-1
*CLDN5*: claudin-5
PUFA: polyunsaturated fatty acids.
