# Supplementary material for: Supplemental Xylooligosaccharide Modulates Intestinal Mucosal Barrier and Cecal Microbiota in Laying Hens Fed Oxidized Fish Oil
Source: Front Microbiol. 2021 Feb 22;12:635333. doi: 10.3389/fmicb.2021.635333 (PMC7937631; doi:10.3389/fmicb.2021.635333)
Supplement: Supplementary file 1 [file Table_1.DOCX]

**Table S1.** Comparison of similarities in cecal microbiota composition among groups by ANOSIM analysis based on unweighted UniFrac distance.

| Treatment^1^ | *R*-value | *P*-value |
| --- | --- | --- |
| FFO vs OFO | 0.309 | 0.002 |
| FFO vs OFO/XOS_400_ | 0.730 | 0.001 |
| OFO vs OFO/XOS_400_ | 0.425 | 0.001 |
| FFO vs OFO vs OFO/XOS_400_ | 0.471 | 0.001 |

^1^ FFO, fresh fish oil diet; OFO, oxidized fish oil diet; OFO/XOS_400_, oxidized fish oil diet + 400 mg/kg xylooligosaccharide.

**Figure S1.** Ileal morphology of laying hens in different groups [hematoxylin and erosion (H&E) staining, 40×]. FFO, fresh fish oil diet; OFO, oxidized fish oil diet; OFO/XOS_200_, oxidized fish oil diet + 200 mg/kg xylooligosaccharide; OFO/XOS_400_, oxidized fish oil diet + 400 mg/kg xylooligosaccharide.


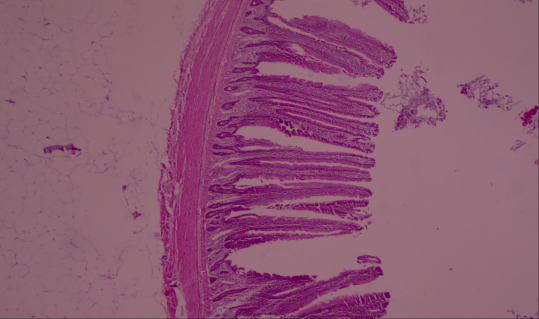

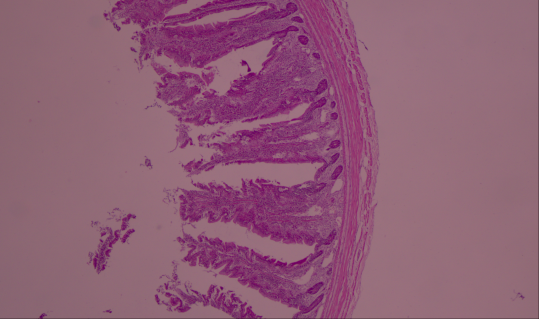

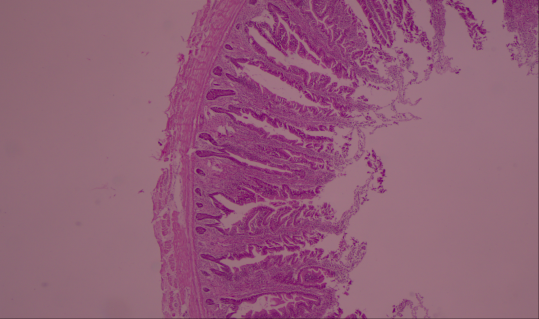

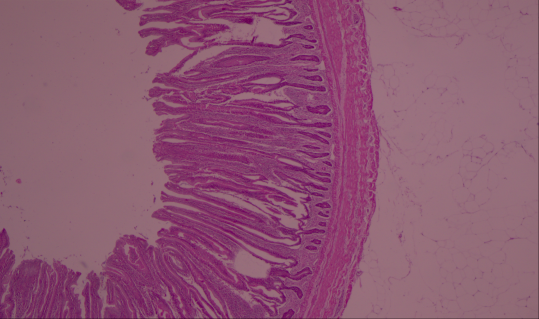


FFO

OFO

OFO/XOS_200_

OFO/XOS_400_

**Figure S2.** Functional comparison of cecal metagenomic sequence based on (A) Clusters of Orthologous Groups (COG) and (B) Kyoto Encyclopedia Genes and Genomes (KEGG) in laying hens (*n* = 8). The differences among the levels of the predicted functions were tested using one-way ANOVA. FFO, fresh fish oil diet; OFO, oxidized fish oil diet; OFO/XOS_400_, oxidized fish oil diet + 400 mg/kg xylooligosaccharide.


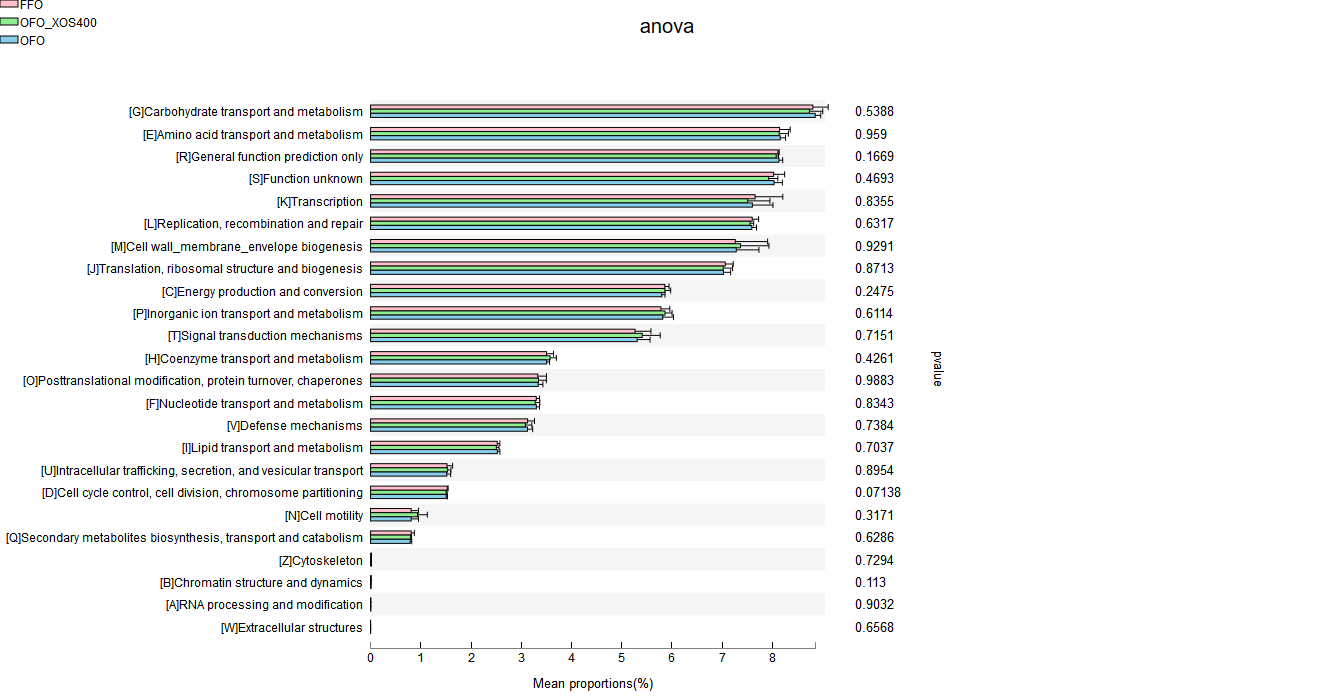


*P*-value

FFO

OFO

OFO/XOS_400_

A


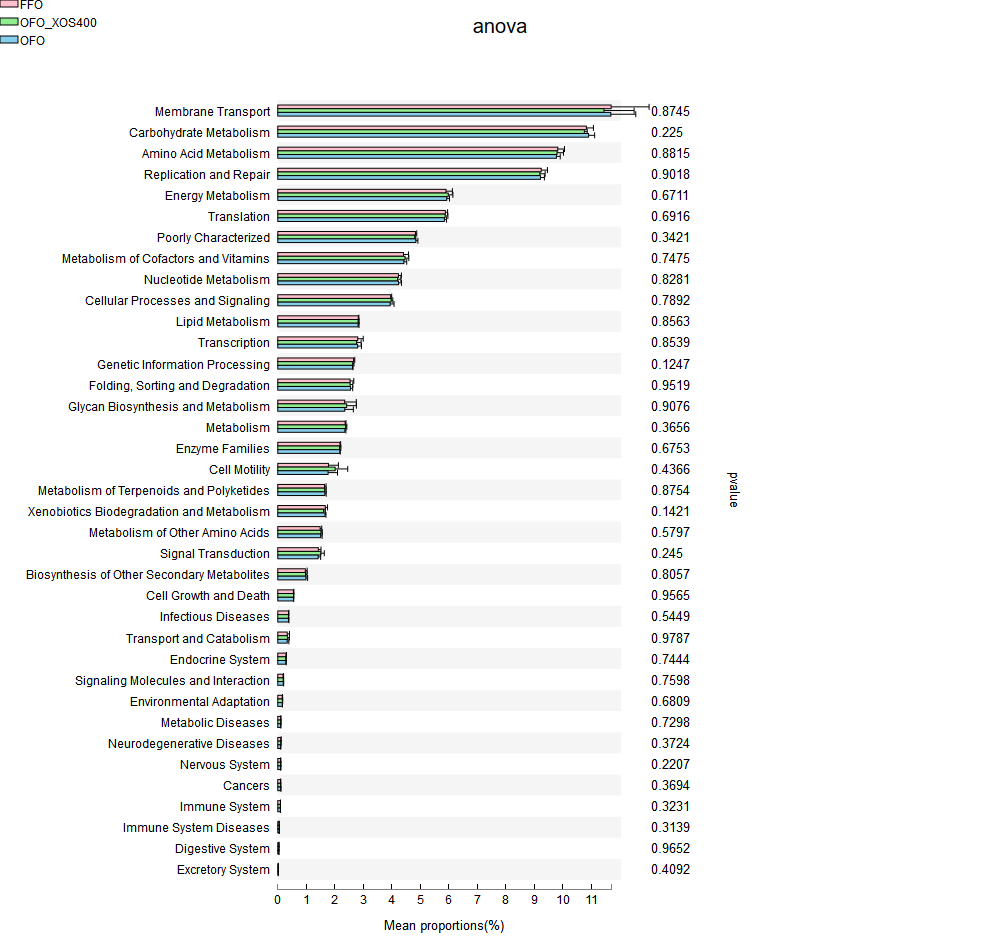


*P*-value

FFO

OFO

OFO/XOS_400_

B
